# Fathers’ views and experiences of their own mental health during pregnancy and the first postnatal year: a qualitative interview study of men participating in the UK Born and Bred in Yorkshire (BaBY) cohort

**DOI:** 10.1186/s12884-017-1229-4

**Published:** 2017-01-26

**Authors:** Z. Darwin, P. Galdas, S. Hinchliff, E. Littlewood, D. McMillan, L. McGowan, S. Gilbody, on behalf of the Born and Bred in Yorkshire (BaBY) team

**Affiliations:** 1 School of Healthcare, University of Leeds, Leeds, LS2 9JT UK; 2 Department of Health Sciences, University of York, York, YO10 5DD UK; 3 School of Nursing and Midwifery, University of Sheffield, Sheffield, S10 2LA UK; 4 Hull York Medical School, University of York, York, YO10 5DD UK

**Keywords:** Perinatal mental health, Paternal mental health, Depression, Anxiety, Screening, Partners, Fathers, Gender

## Abstract

**Background:**

The prevalence of fathers’ depression and anxiety in the perinatal period (i.e. from conception to 1 year after birth) is approximately 5–10%, and 5–15%, respectively; their children face increased risk of adverse emotional and behavioural outcomes, independent of maternal mental health. Critically, fathers can be protective against the development of maternal perinatal mental health problems and their effects on child outcomes. Preventing and treating paternal mental health problems and promoting paternal psychological wellbeing may therefore benefit the family as a whole. This study examined fathers’ views and direct experiences of paternal perinatal mental health.

**Methods:**

Men in the Born and Bred in Yorkshire (BaBY) epidemiological prospective cohort who met eligibility criteria (baby born <12 months; completed Mental Health and Wellbeing [MHWB] questionnaires) were invited to participate. Those expressing interest (*n* = 42) were purposively sampled to ensure diversity of MHWB scores. In-depth interviews were conducted at 5–10 months postpartum with 19 men aged 25–44 years. The majority were first-time fathers and UK born; all lived with their partner. Data were analysed using thematic analysis.

**Results:**

Four themes were identified: ‘legitimacy of paternal stress and entitlement to health professionals’ support’, ‘protecting the partnership’, ‘navigating fatherhood’, and, ‘diversity of men’s support networks’. Men largely described their ‘stress’ with reference to exhaustion, poor concentration and irritability. Despite feeling excluded by maternity services, fathers questioned their entitlement to support, noting that services are pressured and ‘should’ be focused on mothers. Men emphasised the need to support their partner and protect their partnership as central to the successfully navigation of fatherhood; they used existing support networks where available but noted the paucity of tailored support for fathers.

**Conclusions:**

Fathers experience psychological distress in the perinatal period but question the legitimacy of their experiences. Men may thus be reluctant to express their support needs or seek help amid concerns that to do so would detract from their partner’s needs. Resources are needed that are tailored to men, framed around fatherhood, rather than mental health or mental illness, and align men’s self-care with their role as supporter and protector. Further research is needed to inform how best to identify and manage both parents’ mental health needs and promote their psychological wellbeing, in the context of achievable models of service delivery.

**Electronic supplementary material:**

The online version of this article (doi:10.1186/s12884-017-1229-4) contains supplementary material, which is available to authorized users.

## Background

Perinatal mental health refers to mental health in the period spanning pregnancy, childbirth, and the first postnatal year [1]. In this paper we use perinatal mental health as an umbrella term that encompasses mental health problems, psychological distress and psychological wellbeing, consistent with shifts in mental healthcare from solely treating and preventing mental health problems, to also promoting psychological wellbeing [2, 3].

Perinatal mental health problems may be pre-existing (i.e. continuing or recurring in the perinatal period) or new onset [4]. Meta-analyses of studies reporting diagnosed mental health problems and above-threshold symptoms indicate that approximately 5–10% of fathers experience perinatal depression [5] and 5–15% experience perinatal anxiety [6] – about half the rate recorded in mothers [7–9]. Paternal perinatal anxiety and depression can have a profound impact on fathers’ wellbeing, functioning and relationships [10]. Prospective data indicates that their children also face increased risk of adverse behavioural and emotional outcomes and, although maternal and paternal mental illness is modestly correlated, the effects of paternal depression exist after controlling for maternal depression [11, 12]. The costs of paternal perinatal mental illness are currently unknown but likely to be considerable given that the UK’s immediate and long-term costs of maternal perinatal anxiety and depression are estimated at £6.6 billion each year; of which 60% relates to impacts on the child [13].

The positive correlation between paternal and maternal mental health, coupled with early evidence of the emotional, behavioural, and developmental effects that paternal perinatal mental illness has on children, has led to calls for the development of models of service delivery that focus on the psychological wellbeing of the couple and family rather than the individual [5], including the routine assessment or screening of fathers’ mental health and wellbeing in perinatal services [14–16]. Paternal depressive symptoms increase the risk of continued or worsening maternal symptoms [17] and fathers’ positive involvement reduces the risk of adverse behavioural outcomes in the children of depressed mothers [18, 19]. Treating and preventing paternal perinatal mental illness and improving paternal psychological wellbeing therefore not only have benefits for men, but also offer potential to maximise effective support for the mother and child from within the family. Despite this, perinatal services (including mental health services, maternity services and primary care) are currently designed to assess *maternal* mental health and wellbeing, and to prevent and manage *maternal* perinatal mental health problems [20].

In the general population, men are known to manifest their psychological distress differently from women [21]. Men are also less likely than women to access health services relating to their mental health and when they do, tend to use different language, present with different symptoms, and have different needs [22]. However, research on the experiences of mental illness and psychological distress in the perinatal period, how it manifests, its time course, and the barriers and facilitators to seeking and accessing help, has primarily focused on women. Little is known about men’s presentation in the perinatal period and health professionals have described postnatal depression in men as ‘vague and difficult to detect’ [23]. There is a dearth of information regarding men’s experiences of their own perinatal mental health, and our understanding of how best to address fathers’ mental health and psychological wellbeing, including whether they have distinct challenges, needs and preferences, is severely limited as a result [24, 25].

Several reviews have synthesised qualitative research of fathers’ experiences during pregnancy and the transition to parenthood [26–32], but these have examined the broader experiences and challenges encountered by first-time fathers and their experiences of maternity services. The majority of studies addressing men’s experiences and views concerning perinatal mental health have been limited to the experiences of men whose partner has postnatal depression [33–37]. Studies specifically investigating fathers’ own experiences of mental health and wellbeing during the perinatal period are rare.

To the best of our knowledge, to date, only one published study [38] has specifically explored the experiences of fathers with depression. Edhborg et al. [38] interviewed 19 fathers in Sweden who self-identified as having depressive symptoms 3 to 6 months postpartum. Prominent in men’s accounts were deterioration in their relationships with their partners and difficulties in balancing the competing demands of family, work, and their own needs. Fathers in the study experienced themselves as invisible and excluded as parents, and lacked adequate help and support to meet the challenge of new fatherhood and a changed partner relationship.

In light of the lack of evidence, the aim of this study was to examine the views and experiences of first-time and subsequent fathers reporting symptoms across the continuum of psychological distress, concerning their perinatal mental health and explore their perceptions of what makes perinatal mental health resources accessible and acceptable. This research is important to build the evidence base and inform suggestions for service provision.

## Methods

### Design

An interpretive qualitative study using semi-structured interviews with first-time or subsequent fathers.

### Procedure

The Born and Bred in Yorkshire (BaBY) cohort was used as a sampling framework. BaBY (www.bornbredyorks.org) is a population-based prospective cohort of babies and their parents. Parents were originally recruited to the cohort via maternity services at four sites across North Yorkshire and East Lincolnshire in 2011–2014. Partners were invited to take part via the mother, irrespective of their status as the mother’s partner and/or baby’s biological father. Data collected in the BaBY cohort included: maternal background and obstetric history; pregnancy and birth outcomes; and a Mental Health and Wellbeing [MHWB] questionnaire provided to both parents at approximately 26–28 weeks of pregnancy, and 8 weeks following birth. Details of the measures included in the MHWB questionnaire are presented in Table 1.

**Table 1:** Measures contained in the Mental Health and Wellbeing (MHWB) questionnaire

Measure	Description
PHQ-8 [46, 47]	The PHQ-9 is a 9-item questionnaire which assesses the core symptoms of depression and their frequency in the past 2 weeks, rated from 0 (“not at all”) to 3 (“nearly every day”); here, the 8-item version was used, excluding the item on suicidality; cut points of 5, 10, 15 and 20 respectively indicate mild, moderate, moderately severe, and severe levels of depression [46, 47]; cut-off scores between 8 and 11 give acceptable diagnostic properties for detecting major depressive disorder in the general population [48]
GAD-7 [49]	7-item questionnaire which assesses the core symptoms of anxiety and their frequency in the past 2 weeks, rated from 0 (“not at all”) to 3 (“nearly every day”);cut points of 5, 10 and 15 respectively indicate mild, moderate, and severe levels of anxiety [49]; cut-off scores between 7 and 10 give acceptable diagnostic properties for identifying generalised anxiety disorder [50]
PHQ-15 [51]	15-item questionnaire which assesses somatic symptoms (e.g. headaches, dizziness, trouble sleeping) and their frequency in the past 4 weeks, rated from 0 (“not bothered at all”) to 2 (“bothered a lot”); total scores of 5, 10 and 15 respectively indicate low, medium and high levels of somatic symptom severity
LTE (List of Threatening Events) [52]	12-item questionnaire which assesses which of 12 life events (e.g. serious illness, unemployment) have occurred in the timeframe specified

The BaBY research team identified men who met the following eligibility criteria: consented to be contacted again; baby born in the past 12 months at ≥37 weeks’ gestation; no serious health concern with mother or baby before discharge; previously completed mental health and wellbeing [MHWB] questionnaires. Same-sex parents were excluded due to the focus here being fathers. Most babies were aged over 6 months at the time of invitation due to recruitment of parents to the cohort having ceased in 2014.

Eligible fathers were sent a pack containing: a Participant Information Sheet; a pre-paid envelope; a form to record interest in taking part, contact details and consent to access existing BaBY data; and a form to record background characteristics (paternal information was not collected in the original cohort). Reminder packs were sent 2 weeks after initial posting. Those expressing interest were purposively sampled on the basis of their antenatal and postnatal MHWB scores, using maximum variation sampling [39] to ensure that fathers with a range of MHWB scores were included in the study.

### Ethics, consent and permissions

Interviews were transcribed with the principle of anonymity in mind. Participants were able to withdraw from the study, without giving any explanation; none chose to do so. Exemptions to confidentiality were detailed in the consent process; specifically risk of harm to self or others, and instances of possible bad practice by the NHS. Protocols were in place concerning disclosures indicating risk to self or others.

### Sample characteristics

In total, 42 of the 140 eligible men expressed interest in interview, returning their contact details and valid consent to access existing BaBY data. The MHWB scores for men who expressed an interest in taking part in an interview (*n* = 42) and those who did not (*n* = 98) were comparable across all measures and at both time-points (see Additional file 1) and considered representative of fathers in the BaBY cohort. A purposive sample of 22 men with a broad range of MHWB scores were invited for interview, three of which did not respond to the researchers’ efforts to make contact. The participants’ characteristics are shown in Table 2. Mean anxiety and depression scores for interviewees were significantly higher than for those who were not interviewed (see Additional file 2), reflecting the purposive sampling used to provide a sample across the continuum of psychological distress.

**Table 2:** Characteristics of the men interviewed (*n* = 19)

Characteristic	Mean, s.d., range; *n* (%)
Age (years)	33.1, s.d. 5.1 (25–44)
First-time father	
Yes	14 (73.4)
Children from previous relationships	
Yes	0 (100.0)
Age of baby/youngest child (months) at time of invitation	8.1, s.d. 1.5 (5.1–9.8)
Type of birth	
Spontaneous vaginal delivery	11 (57.9)
Caesarean section	5 (26.3)
Instrumental	3 (15.8)
Marital status	
Married	16 (84.2)
Residing together	3 (15.8)
Ethnicity	
White British	18 (94.7)
White – Other	1 (5.3)
UK born	
Yes	17 (89.5)
Employment	
Employed full-time	17 (89.5)
Employed part-time	1 (5.3)
Unemployed	1 (5.3)
Education	
Secondary school	2 (10.5)
Further education (Post-16, including vocational)	7 (36.8)
Higher education (University)	9 (47.4)
Not reported	1 (5.3)
Antenatal mental health and wellbeing scores	
PHQ-8	Mean 3.5, s.d. 2.7, range 0–10
GAD-7	Mean 3.0, s.d. 2.5, range 0–8
PHQ-15	Mean 3.4, s.d. 2.9, range 0–9
LTE (List of Threatening Events)	Range 0–2; 5 scoring ≥1
Postnatal mental health and wellbeing scores	
PHQ-8	Mean 4.8, s.d. 2.8, range 0–11
GAD-7	Mean 4.0, s.d. 2.9, range 0–12
PHQ-15	Mean 4.0, s.d. 2.7, range 0–10
LTE (List of Threatening Events)	Range 0–2; 3 scoring ≥1

### Data collection

Data were collected using semi-structured interviews that followed an interview topic guide (see Additional file 3). All participants were asked the same main questions whilst retaining the flexibility to explore participants’ responses in further detail and change the sequence and phrasing of questions where necessary.

Interviews were arranged at a time and location chosen by the participant and were digitally audio-recorded. Fifteen interviews were conducted face-to-face, either at participants’ homes (*n* = 12) or on University premises (*n* = 3), with the remainder conducted by telephone (*n* = 4). Partners were able to take part in the interview at the discretion of the male participant; five chose couple interviews, requiring informed consent from each parent. Interviews ranged from 18 to 83 min (mean 47, s.d. 18.5). Member checking was conducted during the interview through a process of ‘circling back’ to clarify participants’ interpretation, understanding and meaning using prompts such as “Can you tell me more about that?” “What do you mean when you said, …?”.

### Analysis

Interviews were transcribed verbatim by a confidentiality-bound professional transcription service, then checked for accuracy by the respective interviewer (ZD/PG). Data were managed using NVivo 10 and analysed according to the thematic analysis approach described by Braun and Clarke [40].

Transcripts were independently coded line-by-line by three researchers (ZD, PG, SH) to generate initial codes and search for candidate themes. Coding was an inductive and data-driven process, not informed by an a priori framework. The candidate themes and sub-themes were reviewed and refined in a face-to-face meeting, with input from another researcher (LMc). Additional file 4 summarises the development of the themes, an excerpt of the data analysis audit trail. To help ensure the rigour of the analysis, we undertook a process of peer debriefing, which involved the researchers scrutinising and providing feedback on the appropriateness of each other’s interpretations and searching for disconfirming evidence (deviant cases). Further discussions took place by email, sharing one NVivo file to define and name the themes, and select extracts for inclusion in the paper.

### Reflexive accounting

All researchers involved in the analysis (ZD, PG, SH, LMc) have experience of gender-related qualitative research on sensitive topics and are parents. At the time of the interviews, both interviewers (ZD, PG) had children of similar ages to those babies’ whose parents were interviewed. All of the authors believe that fathers’ mental health is a significant health concern, but hold mixed views concerning the feasibility of routine assessment of fathers’ mental health.

## Results

We identified four major themes pertaining to men’s experiences and views of perinatal mental health:

i) legitimacy of paternal stress and entitlement to health professionals’ support;
ii) protecting the partnership;
iii) navigating fatherhood;
iv) diversity of men’s support networks.

### Theme 1. Legitimacy of paternal stress and entitlement to health professionals’ support

Common across fathers’ accounts was a tacit acceptance of the existence of paternal stress which was often combined with a questioning of their entitlement to having these feelings, and to its recognition by others.

#### Articulating and attributing stress

Men predominantly couched their discussion around ‘stress’ rather than mental health. Descriptions across the time course indicated that their stress during pregnancy had mainly been connected to anxieties around the birth, this was particularly the case for those who had experienced a previous difficult birth. Overwhelmingly, men reported greater stress in the postnatal period, adjusting to the demands of early parenting (specifically, those associated with sleep and feeding) and their altered relationship with their partner. Men’s descriptions centred on ‘role strain’ and ‘role conflict’, noting the loss of their former life and activities – and associated relaxation - and ongoing struggles to balance competing roles and priorities at work and home; struggles that were often compounded by being geographically distant from theirs and their partner’s families.

*I think for me it’s just - the never having any time to relax, it’s just not possible. I’ve got a stressful job then I come home and I tend to get … the tired, stressed baby … I think the stress for me is just the non-stop-ness of it (Father 12)*

Some men made reference to specific stressors; including discrete events (e.g. traumatic birth) and ongoing difficulties (e.g. chronic health of a parent or child); three spoke of financial pressures since becoming parents.

There was a tendency for men to minimise the difficulties that they had experienced and emphasise the challenges faced by their partner, with some questioning the legitimacy of their own mental health needs:

*I’m always conscious that [partner]’s got it a lot worse, so I just sort of get on with it. (Father 2)*

Whereas some men spoke of their partner’s postnatal depression, none used this terminology in describing their own experiences. Two men – both first-time fathers - did however report recent and ongoing depression for which they had seen their GP and been prescribed anti-depressant medication.

#### Symptoms and manifestation

Asked to describe their stress and its nature, men spoke more readily of their cognitive, as opposed to emotional, symptoms of distress. This mainly concerned guilt about being unable to support their partner due to being at work, but some also reflected on their guilt about their feelings, believing that they ‘shouldn’t’ be struggling. Minimising feelings and becoming more irritable with their partner were common reactions to stress, particularly in the early postnatal period.

*I felt guilty actually, guilty going back to work and leaving [partner] with everything. …I was like, I’ve left them all day on their own. I don’t think that’s how she felt but that’s how I felt. (Father 3)*

*I tend to do the typical man thing of hiding it until I can do so no longer. … I’m not the sort to wail and shout and whatever. … I probably just get grumpy and a bit snappy about stuff. That’s pretty much it really. (Father 12)*

More commonly, the men volunteered that they had experienced physical and behavioural signs, including difficulty concentrating at work and suffering with headaches. Men mostly attributed their symptoms to exhaustion, and often found it difficult to disentangle their mental stress from symptoms that were seen to link with fatigue.

*… something physically is going on, on top of the mental stress.... I felt mentally drained as well and tired, but once the physical aspect came into the whole situation as well, that’s when I went to the GP. (Father 19)*

In the majority of couple interviews, disclosures about the psychological and emotional challenges men had experienced were prompted by discussions between partners. For example:

*Partner of Father 6: You went into yourself, I feel.*

*Father 6: Yes, I could feel myself withdraw, so I wouldn’t communicate as much and I would get snappy when sometimes I wouldn’t do. It was something that if I was already close to it, it would be the minutest of things that sometimes would just make me lose it, not lose it, but kind of just [pause]*

*Partner of Father 6: You’d just go off and be very quiet, don’t you, and don’t speak.*

*Partner of Father 7: I don’t think you slept very well [in pregnancy].*

*Father 7: No. I never sleep very well.*

*Partner of Father 7: I don’t know whether I didn’t sleep because I was stressed or because I was pregnant, I don’t know, but I think you didn’t. It probably affected you in that way, didn’t it?*

*Father 7: Yeah. I haven’t slept as well since you got pregnant … It could be worry, or just concerned for her, thinking about the future, about how it would pan out and how we would do things, and just like not being able to switch off properly more than anything.*

It was notable that the two men who reported having consulted their GP in relation to their mental health described more marked symptoms:

*In the end I just couldn’t function… I wasn’t myself. I couldn’t even make simple decisions. (Father 6)*

*I felt so ill, I just wanted to die. I just thought this is awful. (Father 10)*

#### Entitlement to health professionals’ support

Whilst there were examples of men having felt included by health professionals (for example, by being given breakfast on an antenatal ward), many felt excluded and unclear of their own role when in contact with maternity services, particularly at the birth. Men who had described feeling alienated/left out tended to qualify this by emphasising that the focus ‘should’ be on the mother and baby:

*I think at the birth I felt a bit more like a spare part, but then again I mean they were really good with [partner], I just felt in the way sort of thing. (Father 2)*

*[The midwife]’s interested in [partner] and knowing that I was supporting her, but not so much as me, which, they can’t involve everyone, or take a responsibility for everyone … I very much felt like it’s certainly not about me, this. But at the same time, I do very much appreciate the limited resources. They can’t be responsible for everyone. The pregnant woman is the priority, isn’t she. (Father 18)*

Evident in men’s descriptions were feelings of conflict about wanting to be more involved, and a questioning as to whether this may detract from the support provided to women; a situation compounded by limited contact with health professionals and the short, ‘rushed’ appointments often highlighted by men (and their partners, when present at the interview).

*I didn’t feel like I could raise it [questions about previous difficult birth] when I went to the sessions with the midwife beforehand because I thought it was mainly focusing on [partner] and the baby and measurements and all that sort of thing … I think I was thinking, well, [partner]’s pregnant here, they’re focusing on her, I don’t want to sort of drag it out by 15/20 minutes, because of appointments and that, with me asking maybe what seem trivial questions but to me may be important … I was lost there, I suppose. (Father 3)*

Having an unmet need in the context of a perceived under-resourced health service meant that whilst most men were receptive to and often welcomed the suggestion that fathers’ emotional wellbeing might usefully be addressed alongside that of mothers’, in the future, almost all expressed concerns about the impact this may have on existing provision to support women’s mental health. Of note were a minority of men who did not feel the need to ask fathers about their mental health and wellbeing, with one describing it as “surplus to requirements” (Father 17).

*I would be thinking are there going to be the funds to like assist (.) even if I wanted some assistance, how are they actually going to be able to-? There is a shortage of [GPs] nationally so therefore me then going to the GP and saying, you know - if it was affecting me mentally, I’d feel almost like bad about it, I think I’m wasting their time right here, they’ve got people to see who are in more immediate need or something and, you know, so I probably just like hold it in a bit more. (Father 14)*

Men were also conflicted as to which health professional would be best placed to have these conversations, given fathers’ limited contact with services and their perceived role of midwives (both an emphasis on the woman/pregnancy and on physical rather than emotional health). Some also questioned whether men in general would give ‘honest’ answers to questions about their mental health, although emphasised that they themselves would. It was notable that the two fathers who had accessed mental health support in the perinatal period did so via their GPs, not maternity services.

### Theme 2. Protecting the partnership

Central to the majority of men’s accounts were reflections on how their relationship with their partner had evolved during the perinatal period and been affected by their baby’s arrival. Here, men placed great emphasis on their perceived need to protect both their partner, and their partnership. Most spoke about having less time as a couple since their baby’s birth but it was striking that for many men – both those interviewed individually and with their partners - their primary support and confidante continued to be their partner and not friends or other family members. Some men recognised an altered emotional connection with their partners, including feeling more distant. Such comments alluded to having lost some of their own emotional support from their partner; however this was rarely explicitly articulated by men, possibly reflecting their feelings of not being ‘entitled’ to support as indicated in the first theme.

*[our] communications have gone through peaks and troughs. … During her pregnancy we were. I think we probably found ourselves closer actually during it, not that we were far apart before but I think it did… You’ve obviously come together to see this thing growing…. [Now] I probably don’t get the interaction so much with her (Father 13)*

*I think trying to juggle all of that and this child and you know, your relationship is the thing that takes the biggest hit. So I think it’s finding the time…and I think on the surface you probably think you’re okay, because you have a chat when you get in, but … before you know it you’ve not spent any time with each other or spoke to each other … we were probably just not really talking or not interacting with each other, we were just kind of existing … we sort of just never really reconnected … It’s really just facing it, just talk and be honest with them. But you need time to do it and also you need to both be in the same receptive mood. (Father 1)*

The loss of a previous ‘closeness’ was entwined with men’s perceived need to support their partner with her new or altered role, identity, and, for several fathers, her distress and altered mood. Men’s sense of their role as a supporter was overt and often drawn upon when trying to reconcile the legitimacy of their own stress with their feelings that it ‘should’ be the mother who is the focus of supportive care.

*…not being able to see as much of [baby] as I would like to [is] stressful as well trying to - worrying about looking after both of them. … kind of giving [partner] support, as well, with her return to work. She is obviously quite upset about having to leave a 1 year old. (Father 15)*

For most fathers, the parents’ ‘team work’ was viewed as fundamental to coping with the demands of parenting and ‘navigating fatherhood’.

*There’s always been the shared responsibility of if we see the other person getting a bit stressed up by it then the other person takes him away. (Father 13)*

*She’s been very stressed and when she’s stressed I’m stressed, that’s how it goes … I try to make life easy for her and that means I do more and get more. It’s a team. So it’s been a bit fraught but I think we’re kind of coming out of it a bit now. (Father 12)*

Less common were reports of participants struggling to understand their partner’s perspectives and experiences, both physical and emotional, which could be a source of strain in the relationship. Such tensions were apparent in the interviews involving men’s partners, although not necessarily articulated explicitly. Feeling lost as to how to support their partner’s mental health, some men drew on practical approaches and problem-solving strategies:

*I struggled at times because whilst I could see of the physical effects on [partner], I couldn’t understand the emotional and mental effects it was having on her, so I struggled with that, and I probably did become a bit more snappy, definitely low mood at times and struggling to sort of sleep properly, and you have a lot to think about as well so you’re trying to do everything, trying to make sure that we’re ready but also ready with the house and you’ve got so much to sort of think about (Father 6)*

*There’ll be times when she’s positive and absolutely fine and sometimes it goes the other way and she can’t cope and then we just argue …I try to do longer hours, just get up early and go to bed very late and work as hard as I can all the time. (Father 8)*

### Theme 3. Navigating fatherhood

Rather than engage in conversation about how they managed their mental health and wellbeing or accessed support, most men preferred to emphasise what had ‘worked’ in managing stress, focusing on their strategies for bolstering resilience and successfully navigating fatherhood. With the exception of one man (who stated that he had not wanted to be a father), men judged their performance against their aspiration to be a ‘good father’. Being a good father included being a ‘provider’ and ‘protector’ and was seen to extend beyond stereotypical ideals around financial responsibility, to encompass a ‘hands on’ role with their baby and providing effective practical and emotional support to their partner. Thus, for these men, being a ‘good father’ was synonymous with being a ‘good partner’, and ‘protecting the partnership’ (Theme 2) was inherent in navigating fatherhood.

#### Feeling prepared and (changing) expectations

Reflecting on their early perinatal experiences, men spoke of their role in preparing for the birth of their child and for being a parent with examples of reading books, watching TV, talking to others, attending antenatal appointments and antenatal education classes, alongside practical preparations around the house. Most noted however that they had not felt ‘truly’ prepared and parenting only became ‘real’ once they were ‘doing’ it. Whilst this was an observation shared by both fathers and mothers in the couple interviews, the lack of preparation appeared heightened for men, who often noted an absence of information and resources that were tailored and targeted towards them.

In comparing the experiences of first-time fathers and subsequent fathers it was evident that past experience could offer some learning but could also provoke anxiety, such as those concerning the birth.

*We wanted to extend the family, have another child but at the back of my mind I was thinking, oh, we’re going to go through this labour again, which was hell last time, basically. … As we approached due date, I was getting less sleep due to worrying about it, but once it was there, we just got on with it. (Father 3)*

In addition, subsequent fathers faced new challenges in meeting the needs of both children.

*The first time round I didn’t have a clue, the second time round you have a clue but it’s still really scary … Losing the world we had before them was quite scary the first time round, and it hit me quite hard again … your world starts to come back together and then you have another one and it’s harder again because you’ve got two kids and two parents really … so the other one in a way has to kind of entertain [the older child]. (Father 1)*

*You didn’t know how the [older child] was going to react with the new born … it’s not just coping with the pregnancy but it’s coping with explaining to the first (Father 11)*

Most men spoke of the reassurance that they took from coming to have the perspective that many of the challenges were ‘just a phase’; this was particularly voiced by subsequent fathers in comparing their experiences with their younger children. Some men reflected on the importance of changing their expectations, acknowledging that some of their stress reflected an unrealistic standard that they and their partners had set for themselves.

*Even though it wasn’t by the book, but it made our lives a lot easier and that I think helped as well, not listening to what everyone told us (Father 6)*

*We felt this pressure building up the more we read, so that’s why we said, like, just trust our instincts (Father 19)*

#### Managing stress through distraction, denial and release

Taking a practical approach to coping with and managing stress was common in men’s accounts. Some fathers reported finding that the stresses relating to pregnancy and parenting differed to those encountered elsewhere, testing their usual coping strategies and sometimes leaving them feeling powerless:

*I’m probably the sort of bloke who actually just says, ‘oh I’m quite forgetful, so I can forget I’ve had the worst night ever’. I just try and forget it. So that’s probably my coping mechanism. It’s just, trying to forget it and I generally do. And then, I guess, I’ve found in some ways, work quite helpful in that respect, because you can have a crazy night where you have no idea what’s going on with [son’s name], but I can go to work and I feel fine. I’m in control here, I know what to do. There’s people who I can actually communicate with, they’ll do what I ask them to do and vice versa. So I’m probably not the best example, the best person to ask, because I think I just choose to ignore. I’m probably more of an ignorer, which isn’t probably that helpful for [partner]. (Father 18)*

*I think the way I deal with things in general and in work and things, I tend to think quite far ahead. Like I said, I’m quite regimented. So where there’s something like that, like, fatherhood and things that I can’t control and I can’t regiment them and put in a plan and things like that, I find it quite hard to digest. If there’s something I can control, a plan and put in a Gantt chart, great, I can deal with that. And things outside of that, you know, are a bit more chaotic. I find I struggle when I have to think them through a lot and maybe verbalise them with [partner]. (Father 17)*

Where task-oriented strategies to tackle the sources of stress were deemed unavailable or inadequate, men managed their feelings of stress in practical ways, with mixed success. This included using sport as a way to relieve stress, offering distraction and physical release of tension, as well as a form of peer support. Several men spoke of using work as a distraction whereas for others work was a source of stress, limiting their ability to help at home.

*I like my work because it’s technical stuff, I know I can bury myself in it and that will take my mind off it. (Father 7)*

Another, who was unemployed, spoke of housework as a welcome distraction:

*I was just pottering about, just trying to keep occupied with anything I could, the flat was sparkling when she got home, just anything to keep my mind off the fact that I wasn’t with her. (Father 9)*

Whilst most men acknowledged teamwork with their partners and sharing of practical tasks, they often noted that they ultimately resorted to taking a self-reliant and stoical attitude when deemed necessary. Here, perceived expectations of masculinity as well as negative attitudes towards depression were evident in some accounts:

*I’d just get on with it. I would just deal with it myself. That’s what I’ve always done. I think it tends to be a male reaction for most people (Father 8)*

*… there’s always the fear, if you open yourself up and you explain how you are feeling emotionally, like blokes will, sort of, ridicule you … don’t be so airy fairy, you know, that, sort of thing … just because blokes try and act all macho and stuff (Father 14)*

*I am a depressive, I’m depressed right now, have been for a few days. … I don’t think, in any stretch of the imagination, I’m the image of the stereotypical man, and yet I’m never going to be able to break out of the, man up, get on with it thing. And I don’t know where that’s come from, just it’s there. And I think generally, that’s my approach. It’s just a case of head down, battle on through* *(Father 10)*

#### Strength through fatherhood as rewarding

Men’s coping capacity was often strengthened through their positive and rewarding experiences of fatherhood; something that grew with the child’s development and her/his increasing ability to interact:

*I think you cope through him as well, as he gets older. I mean, just smiling to himself and being able to come back and he recognises your face, that kind of stuff is a huge coping strategy. It’s really rewarding, so that makes it worth it. (Father 16)*

*The sleepless nights do take their toll on you, but I don’t know if it’s just the way that I think … but I tend to look at the bigger picture. I just think I’m happy because she’s healthy, she’s smiling… So I think, well, I must be doing something half right for her to be trotting around as she does, and she’s happy with me. (Father 7)*

Fatherhood could also bolster men’s capacity for managing other external stressors:

*But I think as much as having [baby] has caused me to feel more susceptible I think to everything going on, at the same time having him has given me a focus because when I’ve had the energy doing things with him has just taken my mind completely off everything because there’s nothing more sort of enjoyable sometimes than when you’re just doing things with him, whereas other times you just are thinking you just want to switch off and have a bit of break. (Father 6)*

Occasionally men admitted their feelings of rejection or being ‘pushed out’ by the closeness between their baby and partner, and spoke of the importance of having ‘father-baby’ time or activities.

*[For women] it becomes about me and bump, and then me and baby. Whereas fathers, it’s about them, you know, them two over there and me. You feel part of that unit but nonetheless, you’re always separated slightly… that’s just how it is. (Father 10)*

Paternity leave was valued highly, and most men commented that it had not been long enough. Whilst none had chosen to share parental leave some changed their working hours to spend more time with their baby and to relieve their partner from caring for their child. Several men voiced the need to ensure not only time with their baby but also to protect their time, “keeping that family unit strong” (Father 6), again noting the significance of the partnership.

### Theme 4. Diversity of men’s support networks

There was a great deal of diversity in fathers’ support networks, including difference in their relationships and interactions with other men. Whilst a minority spoke of ‘keeping themself to themself’ (coupled with self-reliance), most described a range of people they drew support from, although they varied in the ways that they used this support.

Despite the apparent support networks available to men, their accounts revealed a lack of resources, with several lamenting the absence of tailored and targeted information and some noting the paucity of groups for fathers and male-friendly parenting environments.

#### Pre-existing networks - friends, family and the wider community

Men spoke of existing relationships that offered ways to ‘casually’ explore concerns and gain reassurance, without the need to access formal support. Several men mentioned family - either theirs or their partner’s - who provided reassurance about common complaints and health anxieties with young children. Often this support and advice came from siblings or mothers, rather than fathers, with some men acknowledging the changing involvement of fathers between generations.

*We didn’t know what was wrong [when baby was teething], and I think neither of us was able to reassure the other. But, in those situations, we’ve had other people that have been able to add a bit of a perspective. (Father 4)*

Several men relayed conversations that they had had with other fathers in their wider social networks; including colleagues and neighbours. Sometimes, these were brief conversations in passing, whereas others were ongoing, in-depth discussions. For these men, the discussions allowed them to talk about babies and relationships, alongside topics that did not include these aspects and offered a welcome distraction.

*It was just like, how is your respective other half getting on? … a couple of blokey conversations of, oh she’s doing my head in today, kind of thing … It was just nice just to have that male bonding really, which perhaps my dad and [partner]’s dad didn’t really have. It was almost they had their male friends but they didn’t talk about it… it was nice to have them as a support if you needed or more likely just to have a normal conversation. You didn’t have to be talking about babies and pregnancy and nurseries and everything that goes with it all the time. It was there if you wanted it but you could have a bit of a normalised day to day conversation with them without it. (Father 13)*

*[At work] I can cover an awful lot of different things with them… And in a lot of cases, it is bloke banter. You wouldn’t think that it [but] you’re in the middle of an engineering workshop surrounded by blokes, and we probably spend half the day talking about babies and kids and that sort of thing. But I feel more comfortable with it, because I know that there’s guys there that have had similar experiences or they know what it’s like. They know how I’m feeling if I say, oh, we’ve had a rough night … Some people have had worse experiences, so you think, what we’re going through is normal. (Father 7)*

#### ‘Formal’ peer support and opportunities to meet other fathers

Men were split on their attitudes towards formal peer support and groups. Some discussed their experiences of mother and baby groups and the lack of equivalent groups for fathers.

*I think in some ways it would be helpful before and after to make sure that dads are prepared and that they’re coping and maybe even if it was just away from the mums for some people maybe, because I think some dads might find it a bit embarrassing to sort of say I don’t know what I’m doing. (Father 6)*

Some men described positive experiences of ‘gendered’ events and gatherings with members of their antenatal education classes, after their baby’s birth; however this was limited to experiences of classes accessed privately via the National Childbirth Trust (NCT).

Men’s descriptions indicated that conversations in these groups were characterised as ‘war stories’ and ‘banter’ (Father 16), rather than overtly tackling the emotional aspects of pregnancy and parenting; nonetheless, these could offer venting and validation from peers with similar experiences.

*You know we do talk to each other about parenting stuff but … it’s never a serious conversation, it’s all, done over a beer you know and a few jokes, which is good. (Father 1)*

*If people haven’t got, like I’ve been fortunate enough to know other guys going through a similar thing. I can imagine, yeah, you probably do want to just at least know that you’re not alone. Even if you don’t want to talk about it, just to know, actually, he hasn’t had any sleep either. (Father 18)*

Again, there was evidence of some men feeling conflicted about wanting or needing emotional support.

*I’d feel like I maybe shouldn’t want to want some support, and that I should be fine and I should just get by, and actually I have so did I need it? Probably not. Would it would be nice? Yes, maybe. Would I have gone? Different question again, maybe not. (Father 16)*

*If I’m there and I say, you know, I’m feeling down or whatever they’ll more than likely punch me in the arm and get me a beer and tell me to shut up, which is what I need I think. (Father 17)*

Some men vocalised that they would view fathers’ groups as ‘forced’, questioning their value and preferring to use their own informal support; these pre-existing relationships appeared to enable greater expression and discussion of more sensitive topics, where it was wanted.

#### Lack of information resources tailored to men

There was a common desire among fathers to have more information about pregnancy and parenting, and most expressed a preference for information that was geared towards fathers.

*I wouldn’t have a clue how to go about [accessing groups for fathers]. … with [partner], she can go online and find 28 different chat rooms … I don’t know if those things even exist [for fathers] and I wouldn’t know where to look. (Father 1)*

*Father 3: Yes, what I’m saying is I need pointing in the right direction of going onto MUMbler or whatever.*

*Partner of Father 3: You’d have to join it.*

*Father 3: Well, yes, but as a man, I would not necessarily know to go on to that site to find that information. I wouldn’t even have given it a thought.*

A few men were content accessing existing online resources despite them being oriented towards mothers (e.g. netmums), saying they were accessing it “as a parent” (Father 4); of these one noted not wanting the ‘jokey’ communication used in male arenas.

*I didn’t really get on with the dads’ [forums] … they were quite jokey …I guess I wanted just someone to say, ‘yeah, this is normal, that’s fine, you know, it gets better’, or whatever. (Father 2)*

*I wouldn’t know if there is anything, the equivalent for dads, I’ve not really set out to look that specifically, I’ve just come at it more as being a parent … I absolutely would [feel comfortable using netmums] more than happy to look for help, advice, and other people’s experience anywhere really. (Father 4)*

Many spoke of the value they placed on examples that were based on other men’s experiences, and the need for more information that was not just about being a father but also about supporting their partner – including with their psychological health. Fathers also acknowledged that some men did not feel comfortable accessing classes and recognised that written materials may be more acceptable to some men, offering a route to further information and support, as needed or wanted.

*I really enjoyed reading [the Dads’ handbook] … because a lot of it was based on other people’s experiences so you realise you’re not in the boat by yourself, that there are other people that have been through it and obviously it’s a natural thing that everyone does every day… it made me think about things that I hadn’t thought about or think less about things that I had been thinking about. So that was probably a big thing for me. (Father 6)*

*Perhaps if there was some sort of dads thing, like a bounty pack [information pack provided to mothers in England] which is just for dads. Okay, yeah, it’s got baby stuff in it as well, but it’s baby stuff that the dads would - and I know it’s hard because it shouldn’t matter whether you’re a male or a female really should i … So I don’t think you necessarily want to say right, here is a thing just for dads. But I do think you need something that’s quite directed at them. (Father 1)*

## Discussion

This interpretive qualitative study adds to the limited evidence on men’s experiences and perceptions of paternal perinatal mental health. We sought to examine views and experiences of paternal mental health and wellbeing amongst first-time and subsequent fathers across the continuum of psychological distress, as indicated by self-report measures of depression and anxiety symptoms. The study identified that although men experience psychological distress in the perinatal period they may question the legitimacy of their experiences, foregrounding their partner’s needs. Fathers framed their experiences around stress and navigating fatherhood, central to which was their intra-parent partnership. Whilst men described a diverse range of support networks, evident in their accounts was the paucity of resources that were tailored specifically to men’s information needs and preferences, coupled with some resistance to formal forms of support (e.g. groups).

Aspects of men’s accounts in this study resonated with the themes identified in Edhborg et al.’s (2015) study of fathers with depressive symptoms, specifically those relating to ‘stress’ arising from changes in relationships with partners and balancing the competing demands of family, work, and their own needs. Our findings also cohere with the results of meta-syntheses of qualitative data concerning transitions to fatherhood [26–32] including their aspirations to be ‘good fathers’, and the challenges of the loss of their previous life, altered relationships with their partners, feeling underprepared for birth and parenting, and feeling excluded by services. By including participants who were subsequent fathers, this study identified that while these men may feel better prepared by virtue of their previous experiences, there is potential for paternal concerns surrounding previous perinatal experiences including anxieties based on previous traumatic or difficult birth experiences, which have been largely neglected in the wider literature. This identifies an opportunity where midwives and other health professionals may discuss with both parents their previous birth experiences and address anxieties surrounding future births, alongside birth choices.

Fathers in the current study showed preferences to talk about ‘stress’ (rather than mental illness). This is consistent with the wider men’s health literature [41] and offers parallels with research concerning ‘maternal distress’, which encourages an approach to psychological health that foregrounds the psychosocial context and the challenges experienced in this transition period [42].

The men in this study also showed a preference to focus on their successful navigation of fatherhood. Some men may therefore find materials and resources which promote psychological wellbeing more acceptable if they place emphasis on fatherhood and resilience, i.e. the capacity to cope with adversity, or salutogenesis, i.e. the generation of wellbeing and how to promote health rather than treat illness [43].

The present study substantiates that materials and resources are needed for fathers which recognise their roles (both as a parent and as the mother’s supporter) [44–46] but are also tailored to their needs as men, and aligned with their masculine identities [22, 47]. Useful here is the concept of a partner-oriented masculinity proposed in a longitudinal study of work-sharing couples [48] as this was clearly evident in the men’s accounts in the current study. Relevant too is evidence of gendered approaches to coping, which indicates that men tend to favour problem-focused approaches and women favour emotional-focused approaches [49, 50]. Fathers in this study felt frustration where their default coping strategies were less effective in the context of the perinatal period. Such differences in coping styles and strategies need to be considered both for improving fathers’ psychological wellbeing and in supporting them to provide effective partner support.

By explicitly framing discussions around mental health and wellbeing, our study has generated new insights around men’s questioning of the legitimacy of their mental health needs and their entitlement to support from health professionals in the context of their partner’s needs and a perceived under-resourced health service. The findings echo a Swedish postnatal questionnaire study which concluded that fathers prioritise their partners’ needs and are often “not comfortable when too much interest is focused on their own health and emotional wellbeing” (p.427) [51]. Men in the current study expressed openness to discussion of their wellbeing but voiced concerns that addressing fathers’ mental health needs may compete with meeting the needs of mothers, who they stated ‘should’ be the focus of care.

Tailored resources for men may promote psychological wellbeing and help to prevent deterioration in mental health but some fathers will have mental health needs that require more intensive intervention. Few interventions exist for men [25] and men may feel more comfortable with partner-inclusive interventions that are targeted primarily at women’s mental health, such as those reviewed elsewhere [24]. Support that is couched around the father’s role as supporter and co-parent rather than identifying as themselves being ‘in need’ may be more attractive to men, and particularly if aligned with the evidence that fathers’ positive involvement with infants when mothers are depressed improves treatment outcomes for the mothers with depression [52]. This would cohere with evidence from the wider men’s health literature that suggests men often need to find ‘legitimate’ ways to access health services that do not challenge (western) culturally-dominant masculine ideals embodied by strength, stoicism and emotional control [53]. Such an approach may also be the most feasible in the context of pressures facing services given that, even in high-resource settings, fewer than 50% of women with perinatal depression or anxiety seek help or are identified; only 10–15% of those receive effective treatment [13]. Issues such as these are also relevant when considering the feasibility of assessing fathers’ mental health, both the possibility of universal assessment of all men (mirroring the approach adopted with mothers) and a case-finding approach with men whose partners are known to have perinatal mental health problems. Adopting the latter has been suggested elsewhere [54] and likely offers a more efficient use of resources with maternal perinatal mental health problems being the greatest risk factor for perinatal anxiety and depression in men [55]. However, a targeted approach risks missing men with mental health problems whose partners do not have an identified need.

It is unknown, both for fathers and for mothers, whether mental health assessment would be more acceptable and effective if assessed together or separately. In most of the couple interviews in the current study, descriptions of symptoms and manifestation of stress in both partners appeared to be prompted by partners’ discussions; however there was one example where the father’s description of his anxieties appeared to be closed down by his partner’s comments. In addition, given the language used by men when describing their stress and its manifestation – including physical symptomatology - further research should examine whether male-specific measures of perinatal mental health in the perinatal period are needed [56].

### Limitations

Using the BaBY cohort as a sampling framework offered an efficient design, maximising existing resources which is particularly important given the publicly funded nature of research. The sample was however limited in several ways due to this approach and through the analysis process it was deemed that data saturation had not been achieved. Firstly, fathers were recruited to the cohort via the baby’s mother and contact in this study involved writing to the address provided. All fathers in this study were residing with their partner (and all were their baby’s biological father, although this was not a requirement of participation in the cohort) and it is likely that the centrality of the partner relationship and the aspiration of ‘good father’ being intertwined with ‘good partner’ would be different in fathers who are not in a relationship with their baby’s mother; in addition none of the parents had children from previous relationships. This study has been limited to fathers and male partners due to approaching perinatal mental health from a men’s health perspective; the extent to which these findings reflect role versus gender is unknown and it is acknowledged that there is a dearth of research concerning same-sex parents.

Secondly, fathers were interviewed when their baby was aged approximately 5–10 months, by which time most reported that their lives had become more ‘settled’ (and less stressful) than in the preceding months. Research is needed that uses longitudinal interviews with fathers to determine their changing needs and associated implications for identification and management of paternal mental illness and paternal psychological distress. Thirdly, the cohort lacked diversity concerning ethnicity and socioeconomic background, both of which are relevant to the construction of fatherhood and potentially intersections with views concerning paternal perinatal mental health.

It is encouraging that the men willing to be interviewed did not differ from those identified as eligible in the BaBY cohort on the known characteristics – i.e. the MHWB scores – and it appears that this is a topic of interest to men whose experiences span the continuum of perinatal mental health and wellbeing.

## Conclusions

The perinatal period can be a time of psychological adjustment and role strain for both parents. Paternal perinatal anxiety and depression presents a significant public health concern with implications for men and their families. The current study shows that fathers may feel reluctant and unable to express their support needs or seek help and question the legitimacy of their experiences; an issue that can be compounded by prioritising their partner’s needs and feeling excluded by services which they perceive to be under-resourced. Our findings add support to calls for tailored and targeted resources to be developed for men. We propose developing and evaluating information resources in different modalities (i.e. printed, online) that are made more accessible by being framed around fatherhood and including reference to stress and behaviours (rather than exclusively mental health and ‘symptoms’). Our research indicates that such resources might usefully be aligned with fathers’ family-oriented masculine ideals, emphasising the value of men’s self-care with reference to their role as protector. Further research is needed with a more diverse range of fathers and other stakeholders, to inform how best to identify and manage paternal perinatal mental health needs, in the context of current service provision.

## Additional files

Additional file 1: Mental health and wellbeing scores of men who did (*n* = 42) and did not express interest in interview (*n* = 98). (DOCX 14 kb)
Additional file 2: Mental health and wellbeing scores of men who did (*n* = 19) and did not take part in interviews (*n* = 121). (DOCX 14 kb)
Additional file 3: Interview topic guide. (DOCX 15 kb)
Additional file 4: Development of themes. (DOCX 16 kb)

## Abbreviations

BaBY: Born and Bred in Yorkshire
MHWB: Mental Health and Wellbeing questionnaires
