# Supplementary material for: Fathers’ views and experiences of their own mental health during pregnancy and the first postnatal year: a qualitative interview study of men participating in the UK Born and Bred in Yorkshire (BaBY) cohort
Source: BMC Pregnancy Childbirth. 2017 Jan 26;17:45. doi: 10.1186/s12884-017-1229-4 (PMC5270346; doi:10.1186/s12884-017-1229-4)
Supplement: Additional file 1: — Mental health and wellbeing scores of men who did (n = 42) and did not express interest in interview (n = 98). (DOCX 14 kb) [file 12884_2017_1229_MOESM1_ESM.docx]

**Additional file 1 Mental health and wellbeing scores of men who did (n=42) and did not express interest in interview (n=98)**

|  | **Men expressing interest in interview**  **BaBY** | **n** | **Men not expressing interest in interview** | **n** | **t** | **df** | **p** |
| --- | --- | --- | --- | --- | --- | --- | --- |
| ***Antenatal (approx. 26 weeks’ gestation)*** | | | | | | | |
| PHQ-8 | Mean 2.5 s.d. 2.8 | 42 | Mean 1.7 s.d. 2.2 | 97 | -1.62 | 137 | .107 |
| GAD-7 | Mean 1.8 s.d. 2.1 | 42 | Mean 1.8 s.d. 2.1 | 97 | -.319 | 137 | .750 |
| PHQ-15 | Mean 2.6 s.d. 2.9 | 42 | Mean 2.3 s.d. 2.6 | 97 | -.593 | 137 | .554 |
| ***Postnatal (approx. 8 weeks)*** | | | | | | | |
| PHQ-8 | Mean 3.6 s.d. 3.0 | 42 | Mean 2.7 s.d. 3.0 | 98 | -1.50 | 138 | .137 |
| GAD-7 | Mean 2.8 s.d. 3.0 | 42 | Mean 2.5 s.d. 3.1 | 95 | -.522 | 135 | .603 |
| PHQ-15 | Mean 3.2 s.d. 2.7 | 42 | Mean 3.3 s.d. 3.3 | 95 | .155 | 135 | .877 |
