# Supplementary material for: Fathers’ views and experiences of their own mental health during pregnancy and the first postnatal year: a qualitative interview study of men participating in the UK Born and Bred in Yorkshire (BaBY) cohort
Source: BMC Pregnancy Childbirth. 2017 Jan 26;17:45. doi: 10.1186/s12884-017-1229-4 (PMC5270346; doi:10.1186/s12884-017-1229-4)
Supplement: Additional file 2: — Mental health and wellbeing scores of men who did (n = 19) and did not take part in interviews (n = 121). (DOCX 14 kb) [file 12884_2017_1229_MOESM2_ESM.docx]

**Additional file 2 Mental health and wellbeing scores of men who did (n=19) and did not take part in interviews (n=121)**

|  | **Men interviewed** | **n** | **Men not interviewed** | **n** | **t** | **df** | **p** |
| --- | --- | --- | --- | --- | --- | --- | --- |
| ***Antenatal (approx. 26 weeks’ gestation)*** | | | | | | | |
| **PHQ-8** | Mean 3.5 s.d. 2.7 | 19 | Mean 1.7 s.d. 2.3 | 120 | -3.12 | 137 | .002 |
| **GAD-7** | Mean 3.0 s.d. 2.5 | 19 | Mean 1.5 s.d. 2.3 | 120 | -2.50 | 137 | .014 |
| **PHQ-15** | Mean 3.4 s.d. 2.9 | 19 | Mean 2.2 s.d. 2.6 | 120 | -1.87 | 137 | .064 |
| ***Postnatal (approx. 8 weeks)*** | | | | | | | |
| **PHQ-8** | Mean 4.8 s.d. 2.8 | 19 | Mean 2.7 s.d. 3.0 | 121 | -2.93 | 138 | .004 |
| **GAD-7** | Mean 4.0 s.d. 2.9 | 19 | Mean 2.3 s.d. 3.1 | 118 | -2.15 | 135 | .033 |
| **PHQ-15** | Mean 4.0 s.d. 2.7 | 19 | Mean 3.2 s.d. 3.2 | 118 | -1.08 | 135 | .283 |
