# Supplementary material for: Fathers’ views and experiences of their own mental health during pregnancy and the first postnatal year: a qualitative interview study of men participating in the UK Born and Bred in Yorkshire (BaBY) cohort
Source: BMC Pregnancy Childbirth. 2017 Jan 26;17:45. doi: 10.1186/s12884-017-1229-4 (PMC5270346; doi:10.1186/s12884-017-1229-4)
Supplement: Additional file 3: — Interview topic guide. (DOCX 15 kb) [file 12884_2017_1229_MOESM3_ESM.docx]

**Additional file 3 Interview topic guide**

Introduction:

Introduce researcher and reasons for research

Explain interview will take approximately one hour and be audio-recorded

Confirm participant consent including confidentiality statement

You filled out some mental health and wellbeing questionnaires in the BaBY cohort. These were done during pregnancy and again at about 8 weeks after your baby was born.

How did you find them?

Were you surprised to be asked them?

Do you remember any of the questions?

Did you feel like anything was missing?/unnecessary?

At any point during the pregnancy or since your baby was born have you been asked how you were feeling?

Yes – who by? No - would you have liked to have been? Why (not)?

What do you think about asking fathers about their mental health and wellbeing as part of routine antenatal/pregnancy care?

When do you think is the best time to ask these questions?

Who do you think is the best person to ask these questions?

How have you been during pregnancy, and since your baby was born?

How did this compare with your expectations?

How has this compared with your partner’s experiences?

How have things been with…. home, work, family, friends, being a father, your relationship?

When was the last time you felt stressed? Felt down, depressed, hopeless? Little interest or pleasure in doing things? Felt nervous, anxious, on edge? Unable to stop or control worrying?

What happened?

Was it similar to other times in your life, or different? In what way?

Do you think anyone else was aware of how you were feeling?

Did you ever talk about how you were feeling?

Who with?

What triggered this? What stopped you?

Was it helpful?

Did you see your GP? When would you consider talking to your GP about how you are feeling? Is this something that you have done in the past?

What support did you get when that happened/when you felt like that?

Family / friends / professionals

What was good about that? / What was not so good about that?

Have you used any online resources?

What was good about them? / What was not so good about them?

Have you used any other types of support?

What would make you more likely to use them?

What sort of support/help do you think should be available to fathers?

Would you like to add anything? Is there anything else that you expected I may ask you about?

Summary

Re-cap discussion

Thank and explain how results will be disseminated

Give contact details should participants wish to get in contact at a later time
