# Supplementary material for: Fathers’ views and experiences of their own mental health during pregnancy and the first postnatal year: a qualitative interview study of men participating in the UK Born and Bred in Yorkshire (BaBY) cohort
Source: BMC Pregnancy Childbirth. 2017 Jan 26;17:45. doi: 10.1186/s12884-017-1229-4 (PMC5270346; doi:10.1186/s12884-017-1229-4)
Supplement: Additional file 4: — Development of themes. (DOCX 16 kb) [file 12884_2017_1229_MOESM4_ESM.docx]

**Additional file 4 Development of themes**

| **Initial themes** | **Final themes** | **Exemplar quotes** |
| --- | --- | --- |
| **Origins**  - role change and role strain  - relationship with partner  - parenting  - specific stressors, e.g. birth, family health  **Recognising and communicating paternal stress**  - articulating stress  - comparisons with partner’s experiences  - recognising to self  - recognising to partner and others  - discussing with professionals  **Support and coping**  - focus on resilience, what ‘worked’, practical coping  - partnership / team work  - talking with others  - professional support  - other activities (e.g. work, sport)  - fatherhood as rewarding, importance of time with baby  - preparation to be a father  - building confidence as parent  - lack of resources  **Goals**  - ‘good father’  - ‘good partner’  - ‘strong family unit’  - successful navigation  ***Cross-cutting themes:***  **Legitimacy of paternal stress**  **Entitlement to support**  **Partnership** | **Legitimacy of paternal stress**  - articulating and attributing stress  - symptoms and manifestation  - entitlement to health professionals’ support | *I would be thinking are there going to be the funds to like assist…even if I wanted some assistance, how are they actually going to be able to-? There is a shortage of [GPs] nationally so therefore me then going to the GP and saying, you know - if it was affecting me mentally, I’d feel almost like bad about it, I think I’m wasting their time right here…they’ve got people to see who are in more immediate need or something and, you know, so I probably just like hold it in a bit more. (Father 14)* |
|  | **Protecting the partnership**  - changing relationship  - protecting partner  - team work essential | *I think trying to juggle all of that and this child and you know, your relationship is the thing that takes the biggest hit. So I think it’s finding the time…and I think on the surface you probably think you’re okay, because you have a chat when you get in, but … before you know it you’ve not spent any time with each other or spoke to each other. … we were probably just not really talking or not interacting with each other, we were just kind of existing. … we sort of just never really reconnected. … It’s really just facing it, just talk and be honest with them. But you need time to do it and also you need to both be in the same receptive mood. (Father 1)* |
|  | **Navigating fatherhood**  - feeling prepared and (changing) expectations  - managing stress through distraction, denial, release  - strength through fatherhood as rewarding | *I’m probably the sort of bloke who actually just says, oh I’m quite forgetful, so I can forget I’ve had the worst night ever. I just try and forget it. So that’s probably my coping mechanism. It’s just, trying to forget it and I generally do. And then, I guess, I’ve found in some ways, work quite helpful in that respect, because you can have a crazy night where you have no idea what’s going on with [son’s name], but I can go to work and I feel fine. I’m in control here, I know what to do. There’s people who I can actually communicate with, they’ll do what I ask them to do and vice versa. So I’m probably not the best example, the best person to ask, because I think I just choose to ignore. I’m probably more of an ignorer, which isn’t probably that helpful for [partner/mother’s name]. (Father 18)* |
|  | **Diversity of men’s support networks**  - pre-existing networks (friends, family, wider community)  - ‘formal’ peer support and opportunities to meet other fathers  - lack of information tailored to fathers | *As far as neighbours’ conversations … it was just like, how is your respective other half getting on? I think probably a couple of blokey conversations of, oh she’s doing my head in today, kind of thing ... It was just nice just to have that male bonding really, which perhaps my dad and [partner]’s dad didn’t really have. It was almost they had their male friends but they didn’t talk about it and I suppose previous generations, you know, talk about your feelings, oh you didn’t do stuff like that, but yeah, it was nice to have them as a support if you needed or more likely just to have a normal conversation. You didn’t have to be talking about babies and pregnancy and nurseries and everything that goes with it all the time. It was there if you wanted it but you could have a bit of a normalised day to day conversation with them without it. (Father 13)* |
